# Non-steroidal Anti-inflammatory Drug Consumption in a Multi-Stage and a 24-h Mountain Bike Competition

**DOI:** 10.3389/fphys.2018.01272

**Published:** 2018-09-10

**Authors:** Daniela Chlíbková, Marina Ronzhina, Pantelis T. Nikolaidis, Thomas Rosemann, Beat Knechtle

**Affiliations:** ^1^Centre of Sports Activities, Brno University of Technology, Brno, Czechia; ^2^Department of Biomedical Engineering, Brno University of Technology, Brno, Czechia; ^3^Exercise Physiology Laboratory, Nikaia, Greece; ^4^Institute of Primary Care, University of Zurich, Zurich, Switzerland

**Keywords:** mountain bikers, endurance, anti-inflammatory drug consumption, ibuprofen, pain relief

## Abstract

**Purpose:** Excessive or inappropriate non-steroidal anti-inflammatory drug (NSAID) use during ultra-endurance events could cause potential risk to athletes’ health. Reports on NSAID consumption in mountain bikers or ultra-mountain bikers are scarce. Therefore, the aim of this study was to investigate the prevalence of NSAID consumption immediately before, during and immediately after a mountain bike (MTB) race and to compare NSAID consumption in two different MTB competitions.

**Methods:** This observational study took place at a three-stage MTB race (SMTB) (*n* = 63) and at a 24-h MTB race (24MTB) (*n* = 68), both held in the Czechia in 2017. NSAID consumption was evaluated via self-reported electronic questionnaires.

**Results:** Of all finishers (*n* = 131), fourteen (10%) consumed NSAID at least once during the competition day (immediately before, during or immediately after the race). The number of NSAID consumers was the same in both competitions. Nevertheless, only three athletes (2%), all of them from the 24MTB, consumed NSAID during the race and 5% of all mountain bikers reported consumption after the race. In contrast to the SMTB, the intake reported by the 24MTB participants was quite homogeneous in terms of the timing of NSAID consumption. The NSAID users were older (*p* = 0.043) than the non-users. Ibuprofen was most commonly used by 79% of all consumers.

**Conclusion:** The prevalence of NSAID use was higher in the older participants and seems to be lower in comparison with results from studies about runners, ultra-runners and triathletes suggesting that it is determined by the discipline (i.e., cycling). On the other hand, the timing of NSAID consumption was probably affected by the competition character (e.g., MTBS or 24MTB). Future studies should focus on a larger sample size of cyclists from various disciplines.

## Introduction

Non-steroidal anti-inflammatory drugs (NSAIDs) are commonly used in sports medicine (Paoloni et al., 2009). A high rate of non-prescribed use as well as a limited awareness about their side effects has been reported previously (Gorski et al., 2011). NSAID are one of the most commonly used medications for the treatment of musculoskeletal pain and inflammation in all classes of athletes (Ciocca, 2005). However, the use of these drugs does not always induce a positive effect in athletes. Excessive or inappropriate NSAID use during ultra-endurance events could cause potential risk to the athletes’ health. For instance, it is noteworthy that the use of NSAID did not appear to reduce the development of muscle damage in a 160-km ultramarathon, and, paradoxically, was associated with elevated biochemical markers of inflammation (Nieman et al., 2006). Moreover, NSAID have been indicated as a risk factor in the development of exercise-associated hyponatremia (Davis et al., 2001; Wharam et al., 2006; Page et al., 2007; Warden, 2010) and may subsequently enhance the development of rhabdomyolysis (Bruso et al., 2010). Besides, NSAID may probably affect renal functions by increasing the effect of water retention by arginine vasopressin in kidneys (Baker et al., 2005; Warden, 2010) and reducing glomerular filtration rate (Murray and Brater, 1990).

Given the high level of NSAID consumption in ultra-marathons, the renal system seems to be particularly prone to injury at this type of competition (Wharam et al., 2006; Hoffman and Fogard, 2011). NSAID use during an ultra-endurance race can lead to an impaired muscle recovery from exercise as well as to the inhibition of skeletal muscle adaptation to training (Warden, 2009). NSAID have side-effects, such as asthma exacerbation, gastrointestinal problems, hypertension and other cardiovascular diseases (Paoloni et al., 2009). Nevertheless, the results of the above mentioned and other related studies are often conflicting and further investigation is necessary to determine the potential risks of NSAID use in athletes (Hew-Butler et al., 2015).

From the overview above it is evident that the prevalence of NSAID use in various endurance disciplines has been thoroughly documented in the literature. However, to the best of our knowledge, this topic has not been examined in mountain bike (MTB) disciplines so far. Moreover, only a few studies reported NSAID consumption at multiple timings during endurance events (Joslin et al., 2013; Martínez et al., 2017). There has been an increasing scientific interest for MTB as many mountain bikers participate in races for enjoyment, health and fitness, event status, team, social interaction, relaxation and dedication (Shafer and Scott, 2013). In addition, ultra-endurance MTB, where mountain bikers have to cover distances of more than 100 km and large altitude changes, gets very popular, too (Gloor et al., 2013).

Therefore, the aim of the present study was to evaluate NSAID use by mountain bikers. The 30–47% of long distance triathletes (Wharam et al., 2006; Gorski et al., 2011), 35–75% of ultra-marathon runners (Nieman et al., 2006; Hoffman and Fogard, 2011) and 46% of marathoners (Tillander et al., 2018) were found to ingest NSAID during competition. Similar results (about 48% of the athletes involved in the study) were observed in ultra-endurance mountain running events with various race distances ranging from marathon to ultra-distance of 112 km (Martínez et al., 2017). We assumed therefore a similarity in race and recovery NSAID use in mountain bikers compare to runners and triathletes, because these type of endurance races require relatively homogenous preparation and also recuperation and a possibility of pain experience during the race. Unique data representing NSAID consumption at three different time points – immediately before, during and immediately after the race – were used in this study. Furthermore, for the first time, NSAID consumption was analyzed via data collected in two different MTB competitions differing in their character and duration – a three-stage race and a 24-h race.

## Materials and Methods

### Data Collection

The protocol was in accordance with the Declaration of Helsinki regarding research on human subjects and was approved by the local institutional ethics committee at the Centre of Sports Activities, Brno University of Technology, Brno, Czechia. All competitors (over 18 years of age) were previously informed about the study. Information about a forthcoming post-race survey was included in pre-race e-mail correspondence with entries from race directors of both races. An e-mail that invited their participation in a on-line survey was sent the day after the race. About 10 days later, an attempt was made to contact those who had still not responded. The survey was closed 30 days after the race. Written informed consent was obtained from all research participants. A questionnaire based on a previous study (Martínez et al., 2017) was verified and fine-tuned during a pilot study, conducted in 2016 on fifteen 24-h mountain bikers and ten multi-stage mountain bikers with a varying endurance experience.

The questionnaire included questions covering the following topics: (a) general information about the participants (e.g., sex, age, self-reported weight, height and weekly training load); (b) general information about NSAID consumption (e.g., with the following options: habitual/daily use of NSAID, use on a competition day, before competition, during competition, or after competition, none); (c) timing of NSAID consumption (e.g., with the following options: use before, during or after competition, none); (d) the kind of NSAID used (e.g., with the following options: ibuprofen, aspirin, naproxen, nimesulide, diclofenac, dexketoprofen, salicylic, other, none) and dose in mg; (e) reasons of NSAID consumption (e.g., with the following options: pain prevention, pain relief, injury treatment, other) and (f) prescription (e.g., medical prescription, own decision, suggestion of teammates, other). BMI was calculated using body mass and body height.

### Participants

Sixty-two amateur multi-stage MTB racers (60 men and 2 women) and sixty-eight 24-h mountain bikers (65 men and 3 women), who finished the race, volunteered to take part in the study (23% of the total 276 finishers and 19% of the total 349 finishers, respectively). Basic demographic and training data about all the mountain bikers including their race performance is shown in **Table 1**. Pre-race characteristics of the multi-stage MTB racers show that their age ranged from 31 to 48 years, body mass index (BMI) ranged from 21 to 29 kg/m^2^ and their pre-race training load was at 4–15 h per week. Pre-race characteristics of the 24-h MTB racers show that their age ranged from 37 to 57 years, BMI from 23 to 29 kg/m^2^ and their training load at 4–10 h per week.

**Table 1:** Pre-race characteristics and race performance of mountain bikers.

	Total (*n* = 130)	MTB multi-stage race	(*n* = 62)	24-h MTB (*n* = 68)	*p*-value
Age (years)	37 ± 9	35 ± 8	39 ± 9	0.006
Body mass (kg)	79 ± 10	77 ± 9	82 ± 10	0.003
Height (cm)	181 ± 7	181 ± 6	182 ± 8	0.601
Body mass index (kg/m^2^)	24 ± 2	23 ± 2	25 ± 2	0.0005
Training load (hours/week)	9 ± 4	10 ± 5	8 ± 4	0.029
Performance	–	21 ± 6 h	257 ± 56 km	–

*Expressed as mean ± standard deviation. MTB, mountain biking*.

### Multi-Stage MTB Race and 24-h MTB Race

Two races were monitored: The ‘Kupkolo.cz MTB Trilogy’ multi-stage MTB race and the ‘24-h MTB 2017’ MTB race. The ‘Kupkolo.cz MTB Trilogy’ multi-stage MTB race took place from July 5 to July 8, 2017 in Teplice nad Metují, Czechia. The 4-day race consisted of a prolog and three stages. The prolog covered 8 km with 300 m difference in elevation, stages 1 and 2 covered 70 km each and stage 3 was 80 km long, with approximately 8,400 m of total elevation. The stage routes consisted predominantly of trails, interrupted by only a few road sections. During the race, the average temperature reached 23 ± 4°C and the relative humidity was 54 ± 1%. In this competition, total finishing time (i.e., considering all competitions days) was used as a characteristic representing the competitors’ performance.

As regards the 24-h competition, the data was collected at the 8th edition of the ‘24-h MTB 2017’ MTB race in Jihlava, Czechia. The participants started at 12:00 on May 19 and finished at 12:00 on May 20, 2017. The course comprised a 9.5 km single-track with an elevation of 220 m. The average temperature was 17 ± 3°C and the relative humidity was 76 ± 5% during the race. In this case, the overall distance (in km) covered within 24 h was used to evaluate the performance.

### Statistical Analysis

Statistical analysis was performed using Matlab R2014b (The MathWorks, Inc., United States). Continuous data (basic characteristics) was expressed as mean ± standard deviation, whereas categorical data (questionnaire outputs) was shown as frequency counts and percentages. All data was distributed normally according to the Shapiro–Wilk test (*p* < 0.05). A parametric *t*-test was then used to test the differences in the continuous data among all finishers of the multi-stage MTB race and the 24-h MTB race. Mann–Whitney *U*-test was used to test the differences in the continuous data between non-participants and participants of the study, between NSAID consumers from the MTB multi-stage race and the 24-h race and between all non-consumers and all consumers (*t*-test is not suitable in this case due to the low number of samples). The differences in categorical data between the above data groups were assessed by Fisher’s exact test. In all tests, *p* < 0.05 was considered statistically significant.

## Results

### General Information About Study Participants

When comparing the two data sets (**Table 1**), the 24-h mountain bikers were significantly older, with a higher body mass, a higher BMI and a lower training load 3 months prior to the competition than the multi-stage mountain bikers. Body height was the only characteristic which showed no significant difference between the data sets. The finishing time after the multi-stage MTB race calculated from all study participants’ results ranged from 13 h 28 min to 37 h (with the mean time of 21 ± 6 h) corresponding to 4th to 203rd place in the full list (regarding all 276 finishers). Multi-stage mountain bikers who consumed NSAID completed the race in 22 ± 4 h (ranging from 16 h to 25 h 20 min), corresponding to the 27th–119th place in the full list. The mean distance covered in the 24-h MTB race was 257 ± 56 km and ranged from 158 to 369 km, which corresponds to the 40th–123th place in the overall order (of all 349 finishers). Those who consumed NSAID in the 24-h MTB race covered 222 ± 39 km (ranging from 185 to 265 km), which corresponds to the 66th–109th place in the overall order.

Basic demographic characteristics of study participants were similar to those of non-participants (i.e., those racers, who did not complete the questionnaire). As for multi-stage MTB race, the group of non-participants involved 5% (*n* = 10) and 95% (*n* = 204) of women and men, respectively. In participants of the study, these values were 3% (*n* = 2) and 97% (*n* = 60), respectively (see above). The non-participants’ age ranged from 18 to 59 with mean value of 35 ± 7 years. No significant differences were found in age between multi-stage mountain bakers who participated and did not participate the study (*p* = 0.33). In 24-h MTB race, women and men number in non-participants reached 4% (*n* = 11) and 96% (*n* = 281), respectively. It completely corresponded with the percentages obtained in participants (see above). The age in non-participants ranged from 19 to 70 with mean value of 40 ± 10 years, which was not significantly different from the same characteristic in the participants (*p* = 0.615).

### General Information About NSAID Use

Age, BMI, and pre-race training load of mountain bikers who consumed NSAID are shown in **Table 2**. The number of NSAID consumers in the multi-stage MTB race and in the 24-h MTB race was the same with 10% of all participants. We found no significant difference between the two groups with respect to their age, BMI or training load (*p* > 0.05) (**Table 2**). Pre-race parameters of NSAID consumers and non-consumers are shown in **Table 3**. NSAID users were older than non-users (*p* = 0.043) and there was a non-significant difference in BMI, but on the border of significance (*p* = 0.052) (**Table 3**). Age was not associated with race performance (hours or kilometers) and training load during the 3 months prior to the race (*p* > 0.05) neither in all mountain bikers (*n* = 130), nor in NSAID consumers (*n* = 14).

**Table 2:** Age, body mass index, and pre-race training load of mountain bikers who consumed NSAID.

	Total (*n* = 14)	MTB multi-stage race (*n* = 7)	24-h MTB (*n* = 7)	*p*-value^∗^
Age (years)	43 ± 10	39 ± 8	43 ± 7	0.665
Body mass index (kg/m^2^)	25 ± 3	24 ± 3	26 ± 2	0.138
Training load (hours/week)	9 ± 4	11 ± 4	7 ± 2	0.06

*^∗^Mann–Whitney *U*-test, *p* < 0.05*.

**Table 3:** Pre-race characteristics of consumers and non-consumers of NSAID.

	NSAID non-consumers (*n* = 116)	NSAID consumers (*n* = 14)	*p*-value^∗^
Age (years)	36 ± 9	43 ± 10	0.043^∗^
Body mass (kg)	79 ± 10	80 ± 7	0.709
Height (cm)	182 ± 7	178 ± 8	0.093
Body mass index (kg/m^2^)	24 ± 2	25 ± 3	0.052
Training load (hours/week)	9 ± 4	9 ± 4	0.916

*^∗^Mann–Whitney *U*-test, *p* < 0.05*.

### Information About Timing of NSAID Consumption

Of all participants, 10% reported using NSAID at least once on the competition day (i.e., immediately before, during or immediately after the competition) (**Table 4**). There was no statistical difference regarding the timing of consuming between the multi-stage MTB race and the 24-h MTB race participants (**Tables 4**, **5**). Prior to the race, NSAID consumption was the same in both groups. The 24-h MTB racers used NSAID throughout the whole competition day and most of them reported using NSAID during the race (**Table 5**). On the contrary, multi-stage MTB racers mainly reported NSAID intake after the competition without NSAID use during the race (**Table 5**). None of the participants combined NSAID intake during the competition with the intake before or after the competition.

**Table 4:** Pattern of NSAID consumption.

	Total (*n* = 130)	MTB multi-stage race (*n* = 62)	24-h MTB (*n* = 68)	*p*-value
Habitually^∗^	6 (5%)	2 (3%)	4 (6%)	0.682
Competition day^∗∗^	13 (10%)	6 (10%)	7 (10%)	1.000
Before competition	4 (3%)	2 (3%)	2 (3%)	1.000
During competition	3 (2%)	0	3 (4%)	0.246
After competition	7 (5%)	5 (8%)	2 (3%)	0.257

*Expressed as a number of participants in the corresponding category and as a percentage of the total number of participants in the corresponding category. MTB, mountain biking. ^∗^ indicates daily use of NSAIDs, ^∗∗^ indicates consumption either immediately before, or during or immediately after the race*.

**Table 5:** Distribution of NSAID use in NSAID consumers.

	Total	(*n* = 14)	MTB multi-stage race (*n* = 7)	24-h MTB (*n* = 7)	*p*-value
Before competition	4 (29%)	2 (29%)	2 (29%)	1.000
During competition	3 (21%)	0	3 (42%)	0.192
After competition	7 (50%)	5 (71%)	2 (29%)	0.286

*Expressed as a number of participants in the corresponding category and as a percentage of the total number of participants in the corresponding category. MTB, mountain biking*.

### Types and Doses of NSAID Intake

**FIGURE 1:**
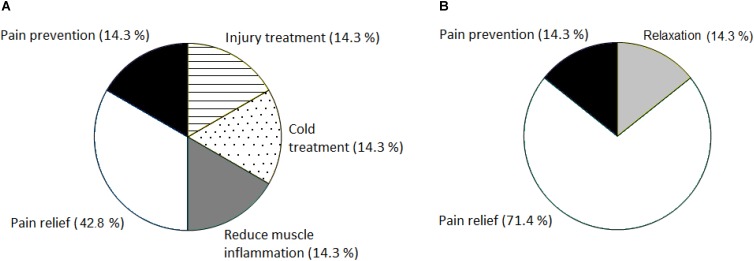
Reasons for NSAID consumption in the MTB multi-stage race **(A)** and in the 24-h MTB race **(B)**. Expressed as a percentage (%) of the total number of consumers in the corresponding category.

Of all the NSAID provided as options in the questionnaire, only ibuprofen, diclofenac and nimesulide were taken by the study participants. The 79% of them reported using only ibuprofen (**Table 6**). Two consumers from the 24-h MTB race reported using diclofenac and one reported using nimesulide. Diclofenac was used before and after the competition, whereas nimesulide was used during the competition (**Table 6**). Multi-stage mountain bikers only used ibuprofen before and after the competition. The average consumption of ibuprofen reached 400–800 mg among the study participants (**Table 7**). Nevertheless, two out of seven multi-stage mountain bikers took 1,600–2,000 mg of ibuprofen (**Figure 1**), which resulted in a higher mean and standard deviation of the analyzed value in this group in comparison with the 24-h mountain bikers (**Table 7**). Despite this fact, no significant difference was found in the ibuprofen consumption between the competitions. Similar results were obtained when taking into the account the duration of the race and expressed the consumption per hour of the competition (**Table 7**). When the two mountain bikers from the multi-stage MTB race with abnormal consumption (see above) were not considered, the average consumption was 27 ± 9 mg/h, which is similar to the consumption in the 24-h MTB group.

**Table 6:** Distribution of ibuprofen and other NSAIDs.

		Total (*n* = 14)	MTB multi-stage race (*n* = 7)	24-h MTB (*n* = 7)
Before competition	Ibuprofen	3 (75%)	2 (100%)	1 (50%)
	Other	1 (25%)	0	1 (50%)
During competition	Ibuprofen	2 (67%)	0	2 (67%)
	Other	1 (33%)	0	1 (33%)
After competition	Ibuprofen	6 (86%)	5 (100%)	1 (50%)
	Other	1 (14%)	0	1 (50%)

*Expressed as a number of participants in the corresponding category and as a percentage of the total number of participants in the corresponding category. MTB, mountain biking*.

**Table 7:** Ibuprofen consumption during the competition day.

	Total (*n* = 11)	MTB multi-stage race (*n* = 7)	24-h MTB (*n* = 4)	*p*-value
Mg	840 ± 548	1000 ± 657	600 ± 231	0.283
mg/hour of competition	39 ± 29	48 ± 35	25 ± 10	0.476

*Expressed as mean ± standard deviation. MTB, mountain biking*.

### Reasons for NSAID Use

The distribution of consumers regarding their reason for NSAID use in both races is shown in **Figure 1**. Obviously, the main reason for NSAID use in both competitions was pain relief, reported by 57% of all NSAID consumers (*n* = 14). About 14% of the consumers from both races used the drugs to prevent pain. Besides above-mentioned options in the questionnaire, other reasons, such as injury treatment, better relaxation and sleep after the race, muscle inflammation reduction or treatment of cold were reported by mountain bikers (**Figures 1A,B**). Only one 24-h MTB participant followed medical prescription for NSAID use. The other participants from both groups (93%) followed their own decision. None of the racers reported using NSAID upon recommendation by a team-mate.

## Discussion

This study is the first to describe the use of NSAID in the context of a MTB race. The aim of this study was to evaluate and compare the prevalence of NSAID consumption immediately before, during and immediately after a MTB multi-stage race and a 24-h MTB race.

### General Information About NSAID Use

The main finding was the predominance of mountain bikers who did not use any NSAID before, during and after the race. Of all the finishers, only 10% consumed NSAID at least once on the competition day (i.e., immediately before, during or immediately after the race). NSAID use during marathon (Joslin et al., 2013; Tillander et al., 2018) and ultra-marathon running (Nieman et al., 2005; McAnulty et al., 2007; Hoffman and Fogard, 2011; Joslin et al., 2013; Martínez et al., 2017) has been previously reported. In a study conducted in 161-km ultra-runners (Hoffman and Fogard, 2011), where NSAID intake was high (about 56% of all study participants), overall drug use was even higher in official finishers than in non-finishers. When we compare running and ultra-running results with ultra-endurance events involving less eccentric exercise, the prevalence of NSAID use was 30% in Ironman triathlon (Wharam et al., 2006) and during a long-distance triathlon the consumption of NSAID reached 47% (Gorski et al., 2011). With regard to the fact that we found no study about NSAID consumption among mountain bikers, the overall NSAID consumption in the present study was relatively low in comparison with runners and triathletes in other studies. The reason could be the lack of typical runners’ medical issues during the race among mountain bikers. For example, blisters as the most commonly encountered medical problem representing 17–40% of injuries at continuous ultra-marathons and 33–74% of injuries during multi stage ultra-marathons; further subungual hematomas, gastrointestinal distress and musculoskeletal injuries representing 1–24% of injuries during continuous and 18–22% during multi stage ultra-marathon running races (Hoffman and Fogard, 2011; Krabak et al., 2011).

The next important finding was that NSAID users were found to be older than non-users. It appears that older athletes had a specific motivation, such as a strong need to finish the race despite pain; however, we did not investigate these psychological factors. Nevertheless, we have to take into account the fact that there may be other factors that we have not studied in this study and which could affect NSAID intake. We can suggest that NSAID intake seems to be lower in comparison with results from studies in runners, ultra-runners and triathletes in the present study.

### Information About Timing of NSAID Consumption

A further important finding was that only 2% from all NSAID consumers used NSAIDs during the race and the 3% used NSAID prior competition. With regard to the timing of NSAIDs, 26% of marathon, half-marathon and marathon relay runners reported NSAID use during competition (Joslin et al., 2013). Even 70% of ultra-marathon runners from the same study were more likely to use NSAID during the 148-mile multi stage race. Twenty percent of 60-km mountain runners used non-selective and 15% selective NSAID before and during the race (Page et al., 2007). The 72% runners consumed NSAID during a 160-km ultra-marathon, (Nieman et al., 2005). During a long-distance triathlon the 47% consumption of NSAID was the day before, immediately before and during the race (Gorski et al., 2011). In a 112-km running race (Martínez et al., 2017), NSAID consumers reported the lowest intake before and the highest intake during the race, while marathoners in the same study reported similar consumption levels before and during the race (Martínez et al., 2017). When we compare the above-mentioned results, we have to take into account the fact that some studies did not exactly clarify if they differentiated between NSAID consumption before and during the race. Nevertheless, a high NSAID consumption during or immediately before a race may put participants at an increased risk of adverse events (Davis et al., 2001; Baker et al., 2005; Wharam et al., 2006; Page et al., 2007; Bruso et al., 2010; Hoffman and Fogard, 2011). With regard to the low NSAID consumption; moreover, consisting from 50% of pre- or during race NSAID intake, the risk of potential medical complications in mountain biker was probably low.

The 5% of mountain bikers reported NSAID use after the race. The marathon runners and ultra-runners both used NSAIDs particularly during recovery and NSAID use was greater in ultra-marathon runners only during the race (Joslin et al., 2013). We confirmed the similarity in recovery NSAID use in mountain bikers compare to runners. Mountain bikers from both races consumed NSAID after the race without significant difference between continuous race – 24-h MTB race and stage race – multi stage MTB race.

### Multi-Stage MTB Versus 24-h MTB NSAID Consumption

A further important finding was the fact that we found no statistical significant difference in NSAID consumption between the group of 24-h mountain bikers and multi-stage mountain bikers. Although the 24-h mountain bikers were significantly older, with a higher body mass and a higher BMI and a lower training load 3 months prior to the competition we found no association between these parameters and the use of NSAID in comparison to NSAID users in the multi-stage race. A potential explanation could be the different numbers in athletes in the two groups. The only difference between the groups was that NSAID consumption was homogeneous throughout the whole competition day in the 24-h MTB racers, whereas the multi-stage MTB racers predominantly used NSAID after the race. These differences could be related to the type of the race, because multi-stage mountain bikers had to complete three difficult stages and they want to regenerate and be ready for the next day. On the contrary, continuous character of the race and its duration probably had an influence on the consumption of NSAID during the race only in the 24-h mountain bikers.

### Types of NSAID Intake and Reasons for NSAID Consumption

Three different kinds of NSAID were consumed in the present study, with the predominance of ibuprofen. It corresponds with the results of Martínez et al. (2017) and Nieman et al. (2005), where it was shown that ibuprofen was taken by 87% and 86% of runners used NSAID, respectively. A large number of athletes take NSAID for their analgesic effects (Nieman et al., 2006; Hoffman and Fogard, 2011). Runners reported NSAID use during competition to help with pain and inflammation (Joslin et al., 2013). Pain relief and the prevention of pain were the most often cited reasons for NSAID consumption in the present study in agreement with previous studies (Gorski et al., 2011; Didier et al., 2017; Martínez et al., 2017). However, no beneficial effects of ibuprofen on decreasing muscle soreness caused by muscle injury were reported (Peterson et al., 2003; Nieman et al., 2005). Moreover, consideration should be taken before ingesting NSAID (ibuprofen) during endurance running, because increased rate of acute kidney injury was reported in those ultra-marathoners who took ibuprofen in a study of Lipman et al. (2017). Renal stress, fluid imbalance and potential acute kidney injury are apparent when cycling in the heat is longer than 5 h and even moderate ibuprofen ingestion had no influence on increased renal biomarkers in road cyclists post-race (McDermott et al., 2018). Although runners prevent muscle soreness caused by eccentric muscle contraction (especially during hill descents) by NSAID consumption, the mountain bikers in our study with predominantly concentric contraction also used NSAID. The main reason for NSAID use in the 24 h mountain bikers was pain relief during and after competition caused probably by the length and continuous load of the 24-h MTB race. The most of multi stage mountain bikers also reported pain relief, followed by pain prevention, injury, cold and muscle inflammation treatment as reasons for prior or after the race NSAID consumption caused probably by demanding repeated stages load. We can assume that older NSAID users who reported more frequently NSAID consumption had probably more medical issues or proven strategy for coping with injury. Tillander et al. (2018) found that runners who reported NSAID use in the past year reported significantly fewer injuries. Anyway, the NSAID consumption was relatively low in the present study. The reason for relatively low NSAID use could be the fact that we examined mountain bikers with recreational approach to sport and they were probably motivated also by personal achievement and general health and were aware of the danger related to drug consumption during a race.

### Limitations, Strength, and Practical Applications

The main limitation of the study is the presence of the biases (such as selection bias, response bias, etc.), which are well known source of the incorrectness in a wide range of study designs, including those based on the questionnaires (Pannucci and Wilkins, 2010). Since, we were conscious of that, particular effort was made in different phases of the study planning and conduction to avoid or at least reduce the biases. Particularly, potential bias sources were considered when designing questionnaire and planning the study. The questionnaire included quite small number of questions, which were phrased well (i.e., with no “sensitive” formulations) and mostly required answering by simple selection from the predefined list to avoid the participant fatigue as well as ambiguous answers. The questionnaire was available in two languages (Czech and English) to reduce number of people, who did not participate in the study due to language barrier, and, consequently, to make the study more representative by including as many people as possible. The racers were informed about a postrace survey via e-mail 1 month before the competition and were additionally invited to the participation 1 day after the race. In both correspondences, no details about the study were included to avoid the affecting of the participants. The online questionnaire was available for 30 days after the competition. Thus, the questions could generally be answered anonymously, independently of other participants, coaches, etc. This time delay might also eliminate the effect of participants’ tiredness present immediately after the race.

During data collection, unfortunately, selection bias cannot be avoided, when people volunteer for a study (so called “self-selection”). There are many methods to reduce selection bias, but the most of them required data from the general population or/and non-participants besides data from the tested group (Keeble et al., 2015). These assumptions are not satisfied in present study due to uniqueness of dataset and the character of the study, where most data are only available for study participants. Further, the participant number could be higher. However, it seems to be similar to that of other studies based on self-reported data focusing on NSAID use (Wharam et al., 2006; Page et al., 2007; Joslin et al., 2013; Martínez et al., 2017). Since the basic demographic characteristics (sex and age) of study participants and the wider population (i.e., the racers, who did not participate) are similar, reported results may be applied – though with a caution – on the general population.

Finally, self-report – a source of response (or reporting) bias – has been the primary method used to assess NSAID use during ultra-endurance events in the field studies. Thus, data should be interpreted with a caution. Nevertheless, confidentiality of data and their representation in a “judgment free” manner, generally “neutral” character of NSAIDs (they are not forbidden or illegal and many people do not know about negative side-effects or positive effects of NSAIDs) eliminated or at least reduced so called “social desirability” effect, which often leads to the reporting bias in self-reporting data.

The findings of the present study were based on data collected through a web electronic survey using similar questions as those of Martínez et al. (2017). Thus, caution would be needed to compare these findings with studies on NSAID prevalence using different methodology.

Despite above aspects, this is the first study of NSAID in MTB athletes, which considering the increasing popularity of this sport would have practical applications for health practitioners and strength and conditioning coaches working with MTB athletes. Medical staff at endurance events need to be aware of potential complications related to eventually excessive use of NSAID in cyclists or athletes in other sport disciplines. Although NSAID use seems to be lower than in runners, ultra-runners and triathletes; a certain percentage of NSAID users has nevertheless occurred also among mountain bikers. Future studies should focus on larger sample size of mountain bikers and also cyclists from different cycling disciplines.

## Conclusion

The prevalence of NSAID consumption was higher in the older participants and seems to be lower in comparison with results from studies in runners, ultra-runners and triathletes suggesting that it is determined by the discipline (cycling). On the other hand, whether NSAID consumption occurs immediately before, during or immediately after the race is probably determined by the competition character (multi-stage competition or 24-h race).

## Author Contributions

DC designed the study, collected all data, and drafted the manuscript. MR, PN, TR, and BK helped in designing the study and drafting the manuscript. MR performed the statistical analyses.

## Conflict of Interest Statement

The authors declare that the research was conducted in the absence of any commercial or financial relationships that could be construed as a potential conflict of interest.
